# Uncovering Indicators of the International Classification of Functioning, Disability, and Health from the 39-Item Parkinson's Disease Questionnaire 

**DOI:** 10.4061/2010/984673

**Published:** 2010-07-12

**Authors:** Maria H. Nilsson, Albert Westergren, Gunilla Carlsson, Peter Hagell

**Affiliations:** ^1^Department of Health Sciences, Lund University, P.O. Box 157, 221 00 Lund, Sweden; ^2^Department of Clinical Nursing Science, School of Health and Society, 291 88 Kristianstad University College, Kristianstad, Sweden; ^3^Department of Neurology, University Hospital, 221 85 Lund, Sweden

## Abstract

The 39-item Parkinson's disease questionnaire (PDQ-39) is the most widely used patient-reported rating scale in Parkinson's disease (PD). However, recent studies have questioned its validity and it is unclear what scores represent. This study explored the possibility of regrouping PDQ-39 items into scales representing the International Classification of Functioning, Disability, and Health (ICF) components of Body Functions and Structures (BF), Activities and Participation (AP), and Environmental (E) factors. An iterative process using Rasch analysis produced five new items sets, two each for the BF and AP components and one representing E. Four of these were found to represent clinically meaningful variables: Emotional Impairment (BF), Gross Motor Disability (AP), Fine Motor Disability (AP), and Socioattitudinal Environment (E) with acceptable reliability (0.73–0.96) and fit to the Rasch model (total item-trait chi-square, 8.28–33.2; *P* > .05). These new ICF-based scales offer a means to reanalyze PDQ-39 data from an ICF perspective and to study its health components using a widely available health status questionnaire for people with PD.

## 1. Introduction

The International Classification of Functioning, Disability, and Health (ICF) provides a conceptualisation and classification of different components of health including biological, individual, and social perspectives [1]. The ICF contains two parts. The first part defines functioning and disability, which in turn consists of two components, Body Functions and Structures, and Activities and Participation. Body functions include physiological and psychological functions, and body structures refer to the anatomical integrity of the body. Activities are the execution of tasks or actions, whereas participation refers to the involvement in life situations. The second part conceptualizes contextual factors, which include environmental and personal factors. The environmental component is the facilitating or hindering impact of the physical, social, and attitudinal environment. Similarly, personal factors are recognized as having a facilitating or hindering impact but they are not further specified because of their vast social and cultural variation [1].

In addition to its use for clinical, educational, and research purposes, the ICF can be used to understand the content of different health outcome measures [2]. Linking such scales to the ICF can be valuable to allow clinical studies to relate to the ICF and for gaining a conceptual understanding of scale contents [3, 4], thereby serving as a base for their further development and refinement. Rules for linking health status measures to the ICF have been proposed and used for linking items of various generic and disease specific scales to the ICF [2, 5, 6]. The findings from such studies can guide researchers and clinicians in their selection of instruments for specific purposes.

The 39-item Parkinson's disease questionnaire (PDQ-39) [7] is the most widely used patient-reported rating scale in Parkinson's disease (PD). However, recent studies have identified problems with its measurement properties. For instance, while the instrument as a whole, as well as its 8-item short form (PDQ-8), appears multidimensional, the validity of the suggested grouping of its items into eight scales also appears questionable [8–10]. Consequently, its validity is at stake as it is unclear what scores represent. Other problems have included suboptimal targeting (items represent more severe health problems than those experienced by people with PD) compromised measurement precision and problems associated with the use of its five response categories [8–10]. Although these experiences point to the need for new developments in patient-reported health outcome measurement for PD, the wide use and spread of the PDQ-39 argues for consideration of alternative and more valid means of using the questionnaire. One way to tackle the validity problems of the PDQ-39 could be to regroup items into theoretically more interpretable domains based on their linkage to the ICF, since this is a universal and standardized nomenclature of functioning and health [11]. Such linking and regrouping of items need to be followed by psychometric analyses and refinement, which preferably is done by means of the Rasch measurement model [12]. 

Here we explore the possibility of regrouping PDQ-39 items according to the ICF framework and test these new scales psychometrically using Rasch analysis.

## 2. Methods

### 2.1. Sample

Details have been reported elsewhere [14]. Briefly, self-reported postal survey PDQ-39 data from 202 people (79% response rate) with neurologist-diagnosed PD were analyzed (Table 1). The sample consisted of people with PD seen at a South Swedish university hospital during one year, excluding those in terminal care and participants in other recent or ongoing questionnaire-based studies. The survey included a question about whether the participant had responded to the questions him-/herself; only respondents who reported that they had answered the survey themselves were included. The study was conducted in accordance with the Declaration of Helsinki, all subjects consented to participation, and the study was approved by the local research ethics committee.

### 2.2. The PDQ-39

The PDQ-39 [7] is a PD specific health status questionnaire comprising 39 items that are grouped into eight scales (Mobility, Activities of Daily Living, Emotional well being, Stigma, Social Support, Cognitions, Communication, and Bodily Discomfort). In addition, an overall PDQ-39 summary index (PDQ-39SI) that summarizes the eight scale scores has been proposed [15]. Respondents are requested to affirm one of five ordered response categories according to how often (from never to always), due to their PD, they have experienced the problem defined by each item during the past month. Higher scores indicate more frequent problems.

### 2.3. Procedure

The 39 items were linked to the ICF by three health science researchers experienced with the ICF and representing different disciplines (nursing, physical therapy and occupational therapy) [2]. First, the 39 items were linked to the most appropriate ICF components (Body Functions and Structures, Activity and Participation, Environment) by each researcher individually. Each item could be linked to more than one component and category [2]. If the information provided by an item was insufficient to allow linkage to an ICF category, the item was considered “not definable”. The researchers discussed their results in two consensus meetings. At the first meeting preliminary consensus were reached. In the second meeting remaining classification difficulties were resolved and items were rearranged into three groups representing the ICF components of Body Functions and Structures (BF), Activities and Participation (AP), and Environment (E).

### 2.4. Data Analysis

Each of the three item groups was individually analyzed psychometrically according to the Rasch measurement model [16] for ordered response categories (the partial credit model) [17, 18].

#### 2.4.1. The Rasch Model

The Rasch model [16, 17, 19] defines, mathematically, what is required from data (item responses) for total scores to express valid measurement. The model is based on the notion that the probability of a certain item response is a logistic function of the difference between the person's location on the measured variable and the level of the variable represented by the item. The model separately locates persons and items on a common logit (log-odd units) metric, with the mean item location set at zero logits. The Rasch model requires unidimensionality (items represent a common underlying latent variable) and local independence (each item response provides unique information). Both these aspects are reflected in the fit of data to the model and violation of either distorts measurement [20, 21]. 

Model fit is assessed by examining the accordance between expected and observed responses across person locations (class intervals) on the measured construct [17, 19]. Overall fit is supported by a nonsignificant item-trait interaction chi-square statistic, and individual item fit is supported by non-significant standardized residuals that range between −2.5 and +2.5 [17, 19]. Residuals represent the discrepancy between observed and expected item responses. Large negative residuals signal local dependency, whereas large positive residuals primarily suggest violation of unidimensionality. 

However, fit statistics can be somewhat insensitive in detecting multidimensionality [22, 23]. Smith [22] therefore proposed conducting a principal component analysis (PCA) of the residuals to identify potential subdimensions in the scale, followed by a series of independent *t* *-*tests to assess whether subsets of items yield different person measures. If violation of unidimensionality is trivial, the number of person locations that differ between the two item sets is small. This approach attempts to assess whether scales are sufficiently unidimensional to be treated as such in practice [22, 24].

The Rasch model also provides a means to assess whether response categories work as assumed [17]. Ordered response categories (e.g., 0-1-2-3-4) are expected to reflect an increasing amount of the variable under investigation. The threshold between two adjacent categories is the point where there is a 50/50 probability of scoring, for example, 2 or 3. Disordered thresholds (e.g., if the 50/50 probability point between scoring 3 or 4 occurs at a lower level of the measured construct than that between scoring 2 or 3) indicate that the response categories do not work as intended. Disordered thresholds may be due to multidimensionality, too many response options, or ambiguous wording. Collapsing categories with disordered thresholds may improve model fit and provide clues regarding how the scale may be improved [25, 26]. 

Differential item functioning (DIF) is an additional aspect of fit to the Rasch model that may result from multidimensionality and can give biased scale scores [19]. DIF analyses assess whether subgroups of people with similar levels on the measured construct respond systematically different to items [27]. When DIF is uniform (i.e., item responses differ uniformly between groups across the measured construct), this can be adjusted for by splitting the item into two new items, one for each subgroup [17]. If this does not improve model fit, the item may be involved in multidimensionality and can be considered for removal.

Targeting assesses how well a scale corresponds to the levels of, for example, health impairments experienced by respondents, by comparing the locations of persons and items. If scales are well targeted to the sample the mean sample location should approximate the mean item location (i.e., zero). Examination of the relationship between the locations of people and item response category thresholds also reveals how successful a set of items are in mapping out a continuum of relevant levels of the measured variable [28, 29]. Targeting also has implications for model fit; when targeting is poor, the ability to assess fit is compromised. Similarly, compromised reliability with poorly separated persons also reduces the ability to detect misfit [17, 19, 28].

#### 2.4.2. Analysis Plan

The overall aim of the analyses was to achieve well fitting and clinically interpretable scales without disordered response thresholds or DIF by gender or age. When analyzing the new item groups the following general approach was therefore taken. First, we deleted not definable items and items that were classified into more than one ICF component. However, since only one item was considered a “pure” environmental item (see Section 3), we combined this with items classified as environmental in addition to tapping either the BF or AP components. 

The resulting item groups were then checked for signs of multidimensionality by means of PCA of the residuals followed by independent *t*-test comparisons of two estimated locations for each person, one based on the items with positive and one from the items with negative residual loadings on the first principal component [24]. Unidimensionality was considered statistically supported if the proportion of significant individual *t*-tests, or the lower bound of the associated 95% binomial confidence interval (CI), did not exceed 0.05 [24]. In case of multidimensionality, items were regrouped according to results of the PCA and theoretical considerations and then analyzed further as separate scales. 

Functioning of response categories was then examined, and if disordered thresholds were found, categories were collapsed. If fit did not improve, items were deleted one at a time, starting with the most misfitting item, while monitoring the resulting overall and item level fit at each stage. The presence of DIF was assessed by comparing item response functions between genders and age groups (as defined by the median, <72 versus ≥72 years old). In case of DIF, these items were split into two new items (one for each subgroup). If this did not improve the scale, the item was deleted. 

The resulting scales were examined regarding reliability and targeting. Reliability was assessed by the person separation index (PSI) [30], which is analogous to coefficient alpha and should exceed 0.7. We also assessed targeting (i.e., how well item locations accorded with the location of the sample) and the extent to which the points of measurement (i.e., the locations of response category thresholds) mapped out an evenly spaced quantitative continuum without significant gaps (indicating compromised measurement ability and larger measurement error) or clustering (indicating item measurement redundancy) [28]. Finally, the logic of the hierarchical ordering of item locations within each scale was considered in order to assess their internal content and construct validity. That is, do item contents appear to represent clinically interpretable variables and is their hierarchical ordering reasonably congruent with increasing and decreasing levels on that variable? 

All analyses were conducted using the RUMM2020 software (Rumm Laboratory Pty Ltd., Perth). Due to the large number of statistical tests, *P*-values were adjusted according to Bonferroni [31].

## 3. Results

Of the 39 items, 30 were judged to belong to only one ICF component (BF, 13 items; AP, 16 items; E, 1 item), eight were judged to belong to two ICF components, and one item was considered not definable (Table 2).

Data quality was good with an average of 2% missing item responses. Rasch analyses of the three item sets showed significant (*P* < .0001) overall misfit with total item-trait interaction chi-square values of 69.12 (BF), 132.36 (AP), and 77.67 (E). Reliabilities were 0.90 (BF), 0.95 (AP), and 0.80 (E). Item level fit statistics are shown in Table 3. PCA followed by independent *t*-tests showed that the proportions of significantly different person measures were 0.15 (95% CI, 0.12–0.18) for the BF items, 0.18 (0.15–0.21) for the AP items, and 0.05 (0.02–0.08) for the environmental items. Based on these findings and conceptual considerations, BF and AP items were then grouped into two subdimensions each (a and b, resp.) before further analyses: BFa (items 17–22), BFb (items 30–33 and 37–39), APa (items 1–7, 11, 12), and APb (items 13–16, 24, 27, 35). The new BF and AP scales displayed improved model fit with total item-trait interaction chi-square values of 10.39 (*P* = .582; BFa), 24.81 (*P* = .036; BFb), 42.24 (*P* = .001; APa), and 28.9 (*P* = .011; APb). Reliabilities were 0.90 (BFa), 0.78 (BFb), 0.96 (APa), and 0.87 (APb). 

**Figure 1:**
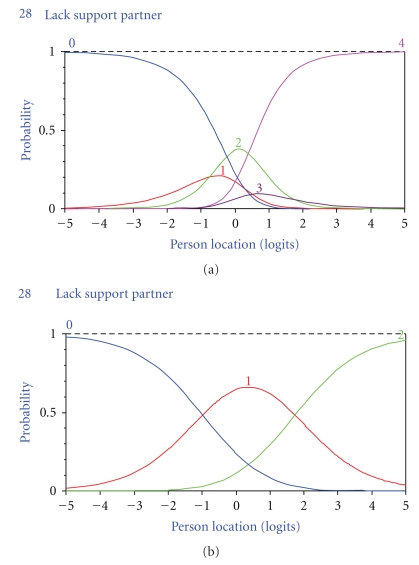
Category probability curves depicting the probability (y-axis) of observing responses in each category (0 = never; 1 = seldom; 2 = sometimes; 3 = often; 4 = always) relative to the location on the measured construct (x-axis; positive values = more problems) for item 28 before (a) and after (b) rescoring. This item was associated with multiple disordering (thresholds 0-to-1/1-to-2 and thresholds 2-to-3/3-to-4) and needed reduction from five to three response categories (combination of responses to categories 1, 2, and 3 into a single category).

Examination of the response categories revealed disordered thresholds in none (BFa and APb), 5 (BFb), 3 (APa) and 4 (E) items. Items 30, 31, 33, 37, and 39 (BFb) and items 3, 11, and 12 (APa) were rescored into four categories (01123). Items 8, 23, 28, and 29 (E) needed to be reduced to three categories (01112). Figure 1 shows an example (item 28) of response category functioning before and after rescoring.

**Figure 2:**
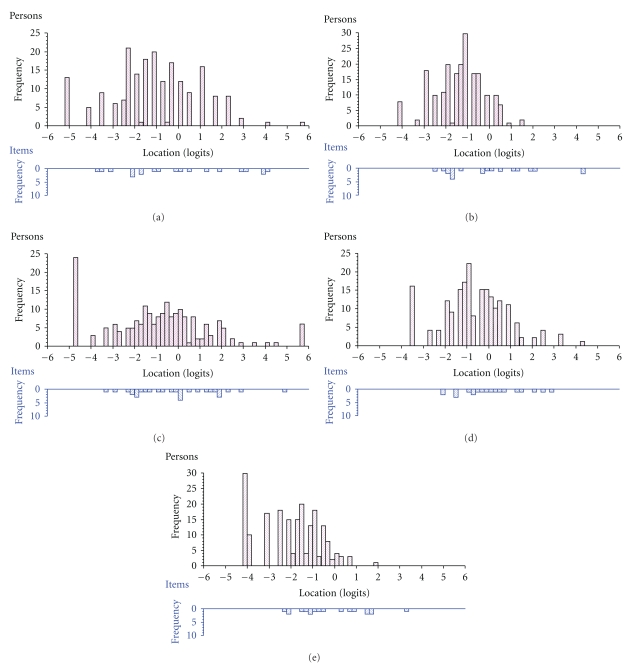
Distributions of the locations of people (upper histogram in each panel) and response category thresholds (lower histogram in each panel) on the common logit metric (positive values = more problems). Response category thresholds are the locations where there is a 50/50 probability of endorsing either of two adjacent categories and represent the quantitative “notches” on the latent ruler defined by a set of items. (a) Item set BFa (items 17, 18, 20–22); (b) Item set BFb (items 30, 32, 33, 37–39); (c) Item set APa (items 1, 2, 4–7, 11, 12); (d) Item set APb (items 13–16, 24); (e) Item set E (items 23, 25, 26, 28, 29, 36). Mean (SD) person locations are BFa (panel (a)), −1.115 (1.951); BFb (panel (b)), −1.363 (1.096); APa (panel (c)), −0.814 (2.326); APb (panel (d)), −0.671 (1.370); E (panel (e)), −1.967 (1.355).

Subsequent stepwise item reduction guided by fit and DIF statistics rendered the new item sets comprising five (BFa) to eight (APa) items each (Table 4). Total item-trait interaction (*P* > .056) and item level fit statistics (Table 4) suggested reasonable fit in all five instances, and reliabilities ranged between 0.73 (BFa) and 0.96 (APa). Figure 2 illustrates the locations of item response category thresholds relative to the locations of the sample for each item set. Inspection of these graphs shows a general tendency for the items (bars below the x-axes) to represent worse health than that experienced by the persons (bars above the x-axes). Furthermore, while the thresholds are able to map out a continuum for each scale, there are also several gaps as well as clusters along those continua (Figure 2).

## 4. Discussion

This study aimed at improving the validity of the PDQ-39 by linking its items to the ICF and to use this as a basis for defining new scales that are more interpretable than the originally proposed eight PDQ-39 scales and its summary index. Results provide support for the notion that this type of exercise is useful in improving the conceptual understanding of health status questionnaires such as the PDQ-39, whose development was not conceptually but primarily data-driven through correlational observations such as factor analysis. Our observations also illustrate that the PDQ-39 can be used to assess the health impact of PD according to the ICF framework by regrouping items and treating them as new scales.

Although the linking procedure employed here means that each of the item sets relate to the respective components of the ICF, these components are in themselves (i.e., without any further specification) relatively unspecific and broad in nature. As such, they only provide a basic framework as to what variables the new PDQ-39-based item sets represent. For the responses to a set of items to be meaningfully summarized into a total score and interpreted as a measure of a common underlying variable, the contents of the items need to express various aspects and degrees of that variable. That is, they should represent clinically reasonable manifestations of the variable and its expressions on a continuum ranging from less to more [28]. Scale construction should therefore preferably begin by defining the variable and its manifestations from less to more; representative items are then generated and selected to cover a relevant range of that variable [32]. However, such a bottom-up approach was not used in developing the PDQ-39 [7] and could therefore not be adapted here either. Instead, it is necessary to consider, based on their contents, what aspects within the respective ICF components the resulting item sets may represent, and if they map out clinically meaningful variables. 

Examination of the item sets and the relative locations of items within each set (Table 4) suggest that four psychometrically valid and clinically interpretable ICF indicators can be inferred from the results of this study, That is, Emotional Impairment (BFa), Gross Motor Disability (APa), Fine Motor Disability (APb), and Socioattitudinal Environment (E). Although the BFb item set exhibited good psychometric properties, it appears unclear what common variable manifestations (items) such as pain, poor memory, feeling unpleasantly hot or cold, and hallucinations would represent. As this item set originates from two of the original PDQ-39 scales (Cognitions, items 30, 32, 33; Bodily Discomfort, items 37–39), it could be suggested to split BFb according to these scales. However, this resulted in considerably reduced measurement precision and reliability (data not shown), and previous studies have shown that these scales are of dubious value according to classical as well as modern test theory analyses [10, 33, 34]. 

Whereas the exact labels of the four suggested ICF indicators may be open for debate, they appear to map out clinically meaningful variables. Table 4 lists each item set according to their locations in the logit metric, where lower values represent less problems relative to items with higher values. This ordering signifies the hierarchical structure of the contents of the variable as manifested by each item set. The hierarchy can therefore be seen as representing the most likely experiences as people progressively move from better to worse health and, similarly, the most probable experiences among people with varying levels of health. As such, it provides a means of judging their clinical feasibility and validity [28, 29, 32]. For example, inspection of the Fine Motor Disability (APb) items suggests that, among the included activities, handwriting is the one that is affected earliest, followed by the ability to do buttons and shoe laces, cut food, and hold a drink without spilling. Finally, at relatively high levels of disability, people avoid eating or drinking in public. This hierarchical pattern seems clinically reasonable and suggests that the items map out various levels of the variable. 

However, it is also evident that the item sets fail to cover the levels of disability experienced by the sample, but tend to represent poorer health. This is reflected by the mean person logit locations (which all are negative), and by the relative distributions of item response category thresholds and persons along the common quantitative continua (Figure 2). This could be due to a sample bias towards people with uncharacteristically mild PD. However, the demographic characteristics of the sample suggest that this is not a major explanation. That is, the respondents appear to represent fairly representative and wide ranges of PD severities (according to Hoehn and Yahr [13] stages), durations and ages. In addition, a majority experienced motor fluctuations, which also speaks against a sample bias towards mild PD. 

In addition to a general bias towards poorer health states, there are also several gaps and clusters of item response category thresholds (see, e.g., the BFa item set; Figure 2(b)). This means that people located around areas associated with gaps are measured with less precision and that differences and changes at these levels will be more difficult to detect [28, 29]. These problems are well known also for the original PDQ-39 [9, 10] and would not be expected to resolve without the addition of new items representing areas not covered by available items [28, 29]. 

When selecting the initial item pool for each of the ICF components, it was decided to use only items that were linked to no more than one ICF component. This decision was made in order to enhance conceptual clarity and interpretability of item sets. However, this strategy could not be pursued for the environmental items since only one item was considered a “pure” environmental item. This means that the resulting Socioattitudinal Environment item set (E) is less specific than the other identified ICF indicators. However, we still believe that these items can be useful as an indicator of socioattitudinal environment in studies wishing to address this component of the ICF, particularly since we are unaware of any other available tool for this purpose in people with PD. 

It may also be argued that Activity and Participation should be separated. However, the ICF provides no clear guidance in this respect. Instead, because of difficulties distinguishing between the two, the ICF offers alternative options for structuring the relationship between them [1, 35], and practices among authors vary [36]. Since the PDQ-39 was not developed according to the conceptual framework of the ICF, it was decided not to separate between activities and participation in this study. Arguably, however, and depending on exact definitions, the vast majority of items linked to this combined AP component (and the resulting scales) appear to represent activity limitations.

As with the original PDQ-39 and PDQ-8 [8–10], the five-category response scale did not work as intended in the new item sets. Although this was compensated for by reducing the number of response categories in the analyses conducted here, it must be emphasized that this exercise is an exploratory post hoc one. Further developmental work and empirical confirmation that reducing and/or rephrasing response categories improve this aspect of the questionnaire is therefore needed.

The PDQ-39-derived ICF indicators identified here do not represent the full ICF spectrum but only limited aspects of its components. For example, it does not offer the possibility to study impairments of body function in terms of the motor symptoms of PD. Furthermore, as with the original PDQ-39 scales, targeting problems prohibit detailed documentation of differences and changes within the respective ICF components, which renders the new item sets relatively coarse. However, this is not likely to be a major problem for their use as survey tools and in other situations where measurement precision may not be of primacy. These limitations of the PDQ-39-derived ICF indicators also point to the need for developing new ICF related tools for use as clinical PD trial outcome measures. Such scales need to be developed from explicit operational definitions of various aspects of the ICF components and should comply with requirements for rating scales to be used as clinical trial outcome measures [37]. Finally, there is a need to reassess the psychometric properties of the PDQ-39-derived ICF indicators in additional samples and cultures in order to establish whether they provide stable and invariant measurement across subgroups of people beyond those studied here.

## 5. Conclusions

This study illustrates that the PDQ-39 can be used to derive psychometrically and clinically acceptable indicators of the main components of the ICF. This provides investigators with a means to reanalyze PDQ-39 data from an ICF perspective and to study its health components using a widely available health status questionnaire for people with PD.

## Figures and Tables

**Table 1:** Sample characteristics (*n* = 202).

Gender (men/women), *n* (%)	108 (53.5)/94 (46.5)
Age (years), Mean (SD; min–max)	69.8 (10.0; 34–90)
Retired, *n* (%)	143 (70.8)
Married or cohabitant, *n* (%)	144 (71.2)
Living in own home, *n* (%)	179 (88.6)
Disease duration (years), Mean (SD; min–max)	8.7 (6.6; 0.5–28)
Hoehn and Yahr stage of PD^a^, Median (q1–q3; min–max)	III (II–IV; I–V)
Perceived disease severity^b^, Median (q1–q3; min–max)	2 (2-2; 1–3)
Motor fluctuations^c^, *n* (%)	137 (67.8)

^a^As assessed for the “off” phase from clinical records. Range, I–V (I = mild unilateral disease; V = Confined to bed or wheelchair unless aided) [13].

^b^Self-rated as mild ( = 1), moderate ( = 2), or severe ( = 3).

^c^Self-reported as present or absent.

PD, Parkinson's disease; SD, standard deviation.

**Table 2:** ICF classification of PDQ-39 items.

Item		ICF classification
No.	Contents (abridged)	
1	Leisure activities	AP
2	Looking after home	AP
3	Carry shopping bags	AP
4	Walking half a mile	AP
5	Walking 100 yards	AP
6	Getting around the house	AP
7	Getting around in public	AP
8	Need company when going out	AP + E
9	Worry falling in public	BF + AP
10	Confined to the house	ND
11	Washing	AP
12	Dressing	AP
13	Do buttons or shoe laces	AP
14	Writing clearly	AP
15	Cutting food	AP
16	Hold a drink without spilling	AP
17	Depressed	BF
18	Isolated and lonely	BF
19	Weepy or tearful	BF
20	Angry or bitter	BF
21	Anxious	BF
22	Worried about the future	BF
23	Felt need to conceal PD	BF + E
24	Avoid eating/drinking in public	AP
25	Embarrassed due to PD	BF + E
26	Worried about people's reactions	BF + E
27	Close relationships	AP
28	Support from partner	AP + E
29	Support from family or friends	E
30	Unexpectedly fallen asleep	BF
31	Concentration	BF
32	Poor memory	BF
33	Distressing dreams or hallucinations	BF
34	Speech	BF + AP
35	Unable communicate properly	AP
36	Felt ignored	BF + E
37	Painful cramps or spasms	BF
38	Pain in joints or body	BF
39	Unpleasantly hot or cold	BF

ICF: International Classification of Functioning, Disability and Health; AP: Activity and Participation; BF: Body Functions and Structures; E: Environment; ND: not definable.

**Table 3:** Rasch item and fit statistics for the initial ICF scales of the PDQ-39^a^.

	Item statistics^b^			Fit statistics^b^		
Component	Item	Location^c^	SE^c^	Residual^d^	Chi square^e,f^	F-statistic^e,g^
BF	17	−0.61	0.09	−1.65	11.30	8.69
	18	0.10	0.08	−1.08	2.70	2.12
	19	0.48	0.09	−0.15	2.39	1.44
	20	0.30	0.09	−0.44	3.11	1.60
	21	−0.27	0.09	−0.99	9.59	5.99
	22	−0.35	0.09	−0.83	7.24	4.47
	30	0.73	0.08	2.98	10.32	4.12
	31	−0.08	0.08	−0.68	4.42	2.82
	32	−0.10	0.08	2.52	2.22	1.19
	33	0.77	0.08	0.70	0.67	0.36
	37	0.01	0.07	2.18	3.80	1.69
	38	−0.64	0.08	1.92	3.62	1.87
	39	−0.33	0.08	2.25	7.76	3.19

AP	1	−0.64	0.09	−0.38	0.56	0.48
	2	−0.47	0.08	−2.06	4.65	4.05
	3	−0.54	0.07	−0.56	0.74	0.26
	4	−0.60	0.07	−0.09	1.11	0.12
	5	0.22	0.08	−0.88	5.21	3.56
	6	−0.18	0.08	−2.45	12.00	10.61
	7	−0.45	0.08	−3.96	18.34	21.79
	11	0.79	0.09	−2.27	10.54	9.84
	12	0.25	0.08	−2.04	5.52	4.67
	13	−0.42	0.08	0.35	4.75	2.47
	14	−0.73	0.09	2.01	12.64	6.26
	15	−0.01	0.08	0.92	2.82	1.72
	16	0.44	0.08	3.74	10.45	3.74
	24	0.48	0.09	2.91	9.05	3.55
	27	1.50	0.10	3.07	31.34	12.40
	35	0.36	0.08	2.27	2.62	1.23

E	8	−0.80	0.06	3.55	34.74	13.72
	23	−0.14	0.08	0.50	1.97	0.83
	25	−0.22	0.08	−0.82	10.90	8.22
	26	0.11	0.09	−0.30	12.94	8.25
	28	0.08	0.10	0.58	4.74	1.68
	29	0.42	0.09	−0.04	5.49	2.38
	36	0.55	0.10	−0.95	6.90	4.04

^a^Performed with the sample divided into three class intervals according to person locations on the measured construct.

^b^Rounded to two decimals.

^c^Expressed in linear log-odds units (logits). Mean item location is zero with positive values representing more health problems.

^d^Residuals summarize the deviation of observed from expected responses. Deviation from the recommended range from −2.5 to +2.5, indicating item misfit, are bold.

^e^Bonferroni corrected statistically significant deviations across class intervals, indicating item misfit, is bold.

^f^Chi-square values summarize the deviation of observed from expected responses across the three class intervals of people. Higher values represent larger deviations.

^g^F-statistics from one-way ANOVAs of deviations from model expectation across the three class intervals of people.

ICF: International Classification of Functioning, Disability and Health; AP: Activity and Participation; BF: Body Functions and Structures; E: Environment; SE: standard error.

**Table 4:** Rasch item and fit statistics for the final ICF scales of the PDQ-39^a^.

Component	Items^b^		Item statistics^c^		Fit statistics^c^			Total item-trait	PSI^h^
	No.	Content (abridged)	Location^d^	SE^d^	Residual^e^	Chi square^f^	F-statistic^g^	Chi-square (*P*-value)	
BFa	17	Depressed	−0.65	0.11	−1.16	2.04	1.61	12.46 (0.41)	0.73
	22	Worried about the future	−0.31	0.11	0.53	0.77	0.39		
	21	Anxious	−0.16	0.12	−0.74	1.88	1.44		
	18	Isolated and lonely	0.42	0.10	0.52	0.51	0.18		
	20	Angry or bitter	0.70	0.11	1.62	1.00	0.58		

BFb	38	Pain in joints or body	−1.02	0.08	−0.21	0.29	0.18	6.15 (0.802)	0.88
	32	Poor memory	−0.46	0.09	0.94	1.04	0.62		
	39	Unpleasantly hot or cold	−0.46	0.11	−0.15	2.21	1.31		
	37	Painful cramps or spasms	−0.06	0.10	−0.63	5.28	3.72		
	30	Unexpectedly fallen asleep	0.95	0.12	2.06	2.26	1.05		
	33	Distressing dreams or hallucinations	1.05	0.12	0.13	1.38	0.82		

APa	1	Leisure activities	−0.93	0.11	2.23	8.81	3.83	33.2 (0.007)^i^	0.96
	4	Walking half a mile	−0.79	0.09	0.59	2.16	1.34		
	7	Getting around in public	−0.62	0.10	−2.36	3.43	3.51		
	2	Looking after home	−0.60	0.10	0.92	2.47	0.93		
	6	Getting around the house	−0.24	0.10	−0.57	2.22	1.44		
	5	Walking 100 yards	0.45	0.10	−0.23	7.40	4.24		
	12	Dressing	0.78	0.14	1.03	0.79	0.28		
	11	Washing	1.95	0.14	−0.54	5.91	3.64		

APb	14	Writing clearly	−0.77	0.09	−0.30	1.54	1.13	8.28 (0.60)	0.86
	13	Do buttons or shoe laces	−0.40	0.08	0.84	0.51	0.19		
	15	Cutting food	0.01	0.09	−0.41	3.69	2.94		
	16	Hold a drink without spilling	0.56	0.09	0.65	2.01	0.97		
	24	Avoid eating/drinking in public	0.59	0.09	1.41	0.54	0.20		

E	25	Embarrassed due to PD	−0.82	0.09	−1.27	1.86	1.27	13.42 (0.34)	0.82
	26	Worried about people's reactions	−0.41	0.10	−1.64	2.85	2.32		
	23	Felt need to conceal PD	−0.20	0.16	−0.05	1.92	1.15		
	36	Felt ignored	0.12	0.10	0.97	3.79	1.98		
	28	Support from partner	0.38	0.18	1.25	2.73	1.28		
	29	Support from family or friends	0.92	0.17	1.55	0.28	0.10		

^a^Performed with the sample divided into three class intervals according to person locations on the measured construct.

^b^Listed in location order. Item numbers refer to the original PDQ-39 questionnaire. A total of nine original PDQ-39 items (numbers 3, 8, 9, 10, 19, 27, 31, 34, and 35) were excluded.

^c^Rounded to two decimals.

^d^Expressed in linear log-odds units (logits). Mean item location is zero with positive values representing more health problems.

^e^Residuals summarize the deviation of observed from expected responses.

^f^Chi-square values summarize the deviation of observed from expected responses across the three class intervals of people. Higher values represent larger deviations.

^g^F-statistics from one-way ANOVAs of deviations from model expectation across the three class intervals of people.

^h^Reliability index analogous to Cronbach's alpha.

^i^ *P* = .057 following Bonferroni correction.

ICF: International Classification of Functioning, Disability and Health; AP: Activity and Participation; BF: Body Functions and Structures; E: Environment; SE: standard error; PSI: person separation index.
